# Supplementary material for: Serum 5-Hydroxyindoleacetic Acid and Ratio of 5-Hydroxyindoleacetic Acid to Serotonin as Metabolomics Indicators for Acute Oxidative Stress and Inflammation in Vancomycin-Associated Acute Kidney Injury
Source: Antioxidants (Basel). 2021 Jun 2;10(6):895. doi: 10.3390/antiox10060895 (PMC8228749; doi:10.3390/antiox10060895)
Supplement: Supplementary file 1 [file antioxidants-10-00895-s001.zip › antioxidants-1220090-supplementary.pdf]

Table S1. Clinical history and characteristics of 7 subjects in the exploratory metabolome profiling analysis

Number	Group	Age/Sex	BMI (kg/m^2)	SCr (mg/dL)	GFR (mL/min/1.73m^2)	CRP (mg/dL)	Vancomycin C_{min} (μg/mL)	Vancomycin treatment indication	Medical history
1	VAKI	70/M	25.3	2.28	31.0	6.02	21.24	Blood stream infection	S/P Heart transplantation status d/t ischemic cardiomyopathy
2	VAKI	69/M	17.1	1.39	51.3	12.36	22.21	CSF infection	S/P Ventriculo-peritoneal shunt due to hydrocephalus
3	infection	56/M	26.6	0.93	89.1	6.10	10.34	Prophylaxis	Subarachnoid hemorrhage
4	infection	52/M	29.0	0.58	124.6	10.55	10.27	MRSA infection in tissue	Chronic otitis media
5	HC	56/F	20.4	0.57	104.0	0.04			Health examination
6	HC	66/M	27.2	0.90	88.7	0.05			Health examination
7	HC	61/F	22.8	0.80	79.8	0.04			Health examination

Abbreviations: VAKI, vancomycin-associated acute kidney injury, HC = healthy control; BMI, body mass index; SCr, serum creatinine; GFR, glomerular filtration rate; CRP, C reactive protein; C_{min}, trough concentration; MRSA. methicillin resistance *Staphylococcus aureus*

Table S2. Comparison of concentrations of serum amino acids and amino acid derivatives correlated with glomerular filtration rate between VAKI group and non-VAKI group

Category	Parameters (μmol/mL)	Correlation with GFR		Decreased GFR groups			Normal GFR groups			
		ϱ^a	*Sig.*[b]	VAKI group	CKD subgroup	*P* [c]	infection subgroup	*P* [c]	HC subgroup	*P* [c]
	Leucine Median (IQR)	0.352	< 0.001	80.2 (59.3 - 99.1)	53.1 (34.5 - 105.9)	N.S	103.1 (87.4 - 140.8)	0.015	106.7 (81.6 - 147.4)	0.006
BCAA	Valine	0.390	< 0.001	129.3 (114.9-160.5)	91.9 (51.4 - 140.8)	0.009	178.1 (138.0 - 222.2)	0.034	156.9 (125.5 - 256.8)	0.014
	Isoleucine	0.290	0.004	32.0 (25.8 - 39.0)	24.4 (16.4 - 40.7)	N.S	41.0 (31.1 - 59.0)	0.041	37.9 (29.2 -53.1)	N.S
	All-isoleucine	0.027	N.S	0.0 (0.0 - 0.0)	0.0 (0.0 - 0.9)	0.007	0.0 (0.0 - 0.0)	N.S	0.8 (0.0 - 1.4)	< 0.001
	Serine	0.357	< 0.001	78.2 (69.2 - 93.9)	63.3 (40.8 - 82.7)	0.021	100.3 (86.7 - 141.9)	0.010	111.0 (75.1 - 1843.4)	0.009
	Methionine	0.263	0.010	6.0 (5.1 - 8.9)	8.0 (4.6 - 15.9)	N.S	9.3 (5.9 - 15.9)	N.S	34.7 (26.9 - 40.0)	< 0.001
	O-phosphoserine	0.240	0.018	1.6 (1.2 - 2.0)	2.4 (1.8 - 3.5)	0.009	3.8 (1.9 - .5.1)	0.002	3.4 (1.6 - 8.3)	0.002
SCAA	Taurine	0.268	0.008	40.6 (32.6 - 50.4)	34.7 (29.1 - 73.9)	N.S	69.8 (39.9 - 109.3)	N.S	81.0 (60.8 - 127.1)	< 0.001
	Cystine	-0.160	N.S	3.7 (1.9 - 8.1)	10.4 (5.2 - 29.4)	< 0.001	11.4 (7.8 - 13.5)	< 0.001	1.2 (0.0 -2.0)	< 0.001
	Cystathionine	-0.236	0.021	0.0 (0.0 - 0.0)	0.8 (0.0 - 1.6)	0.002	0.0 (0.0 - 0.0)	N.S	0.6 (0.0 - 0.5)	N.S
	Anserine	0.483	< 0.001	0.0 (0.0 - 0.0)	0.0 (0.0 - 0.0)	N.S	0.5 (0.0 - 0.8)	< 0.001	0.6 (0.0 - 0.9)	< 0.001
CAA	Carnosine	0.136	N.S	0.0 (0.0 - 0.0)	0.0 (0.0 - 0.0)	N.S	0.0 (0.0 - 0.3)	0.017	0.0 (0.0 - 0.0)	0.039
	Beta-alanine	0.221	N.S	2.1 (1.4 - 2.9)	3.5 (2.4 - 6.5)	0.010	7.3 (6.2 - 8.6)	< 0.001	2.4 (1.9 - 4.7)	N.S

	1-methylhistidine	0.314	0.002	7.4 (5.9 - 9.9)	6.3 (4.1 - 10.1)	N.S	13.6 (10.1 - 14.9)	< 0.001	8.8 (6.2 - 10.3)	N.S
	3-methylhistidine	-0.467	< 0.001	4.8 (4.0 - 7.2)	5.7 (4.2 - 12.3)	N.S	1.7 (0.9 - 10.7)	0.018	2.1 (1.6 - 4.3)	< 0.001
	Arginine	0.144	N.S	72.8 (59.2 - 84.2)	37.3 (23.2 - 59.1)	< 0.001	62.0 (40.6 -143.2)	N.S	73.7 (59.4 - 117.9)	N.S
UCAA	Arginiosuccinic acid	0.303	0.003	0.0 (0.0 - 0.0)	0.0 (0.0 - 0.6)	0.002	0.6 (0.0 - 1.3)	< 0.001	0.0 (0.0 - 2.0)	< 0.001
	Citrulline	-0.065	N.S	35.0 (29.3 - 39.4)	14.1 (9.8 - 22.0)	< 0.001	17.6 (11.2 - 39.5)	0.002	27.4 (22.4 - 33.0)	0.007
	Homocitrulline	-0.360	< 0.001	1.1 (0.7 - 1.5)	0.0 (0.0 - 0.8)	0.001	0.0 (0.0 - 0.4)	< 0.001	0.0 (0.0 - 0.7)	< 0.001
	Alpha-aminobutyric acid	0.475	< 0.001	10.2 (8.2 - 13.4)	6.4 (3.9 - 11.5)	0.037	14.9 (11.4 -19.1)	0.002	16.6 (11.7 - 20.0)	< 0.001
ABA	Gamma-aminobutyric acid	0.441	< 0.001	0.0 (0.0 - 0.2)	0.9 (0.5 - 1.9)	< 0.001	1.9 (1.3 - 2.5)	< 0.001	1.3 (1.1 - 1.6)	< 0.001
	Beta-aminoisobutyric acid	-0.455	< 0.001	2.8 (1.4 - 4.8)	2.6 (0.0 - 9.8)	N.S	0.5 (0.0 - 1.7)	0.001	0.7 (0.0 - 1.1)	< 0.001

Notes:a Each Spearman's correlation coefficient was calculated between GFR and amino acid concentration among total of 117 subjects.

b A two-tailed subsequent significance for correlation coefficient with GFR

c Each *P*-value for comparison of medians of parameters was calculated using the Mann–Whitney U test.

Abbreviations: VAKI, vancomycin-associated acute kidney injury; GFR, glomerular filtration rate; CKD, chronic kidney disease; HC, healthy control; IQR, interquantile range; Sig., significance; *P*, *P*-value; BCAA, branched chain amino acid; SCAA, sulfur containing amino acid; CAA, carnosine associated amino acid; UCAA, urea cycle associated amino acid; ABA, aminobutyric acid

Table S3. Comparison of serum tryptophan, serotonin, and 5-HIAA concentrations from non-VAKI subgroups, including an infection subgroup, CKD subgroup, and healthy control subgroup

Parameters	non-VAKI group				*P*-value[a]		
	Total non-VAKI group	Infection subgroup (1)	CKD subgroup (2)	HC subgroup (3)	1 vs. 2	1 vs. 3	2 vs. 3
Trp [Median (IQR)] (μmol/mL)	25.0 (13.0 - 36.9)	18.9 (12.2-37.0)	14.3 (10.8 - 27.2)	35.4 (25.0 - 47.8)	N.S	0.017	< 0.001
5-HT (ng/mL)	251.2 (114.5 - 389.3)	87.5 (50.6-250.2)	337.6 (195.8 - 520.0)	345.2 (218.4 - 386.4)	0.011	0.015	N.S
5-HIAA (ng/mL)	84.5 (36.3 - 154.5)	80.8 (35.2-258.7)	85.7 (70.4 - 111.8)	38.5 (27.7 - 171.2)	N.S	N.S	N.S
5-HT/Trp	9.8 (3.8-18.0)	4.3 (1.6-16.2)	20.0 (8.6-56.9)	9.4 (4.8-12.1)	0.017	N.S	N.S
5-HIAA/5-HT	0.4 (0.2 - 0.9)	0.9 (0.5-1.9)	0.2 (0.2 - 0.5)	0.3 (0.1 - 0.7)	N.S	0.016	N.S

Notes: a Each *P*-value for comparison of medians of parameters was calculated using the Mann–Whitney U test and adjusted by Bonferroni correction.

Abbreviations: VAKI = vancomycin-associated acute kidney injury, CKD = chronic kidney disease, HC = healthy control, Trp = tryptophan, 5-HT = serotonin, 5-HIAA = 5-hydroxyindoleacetic acid, IQR = interquantile range

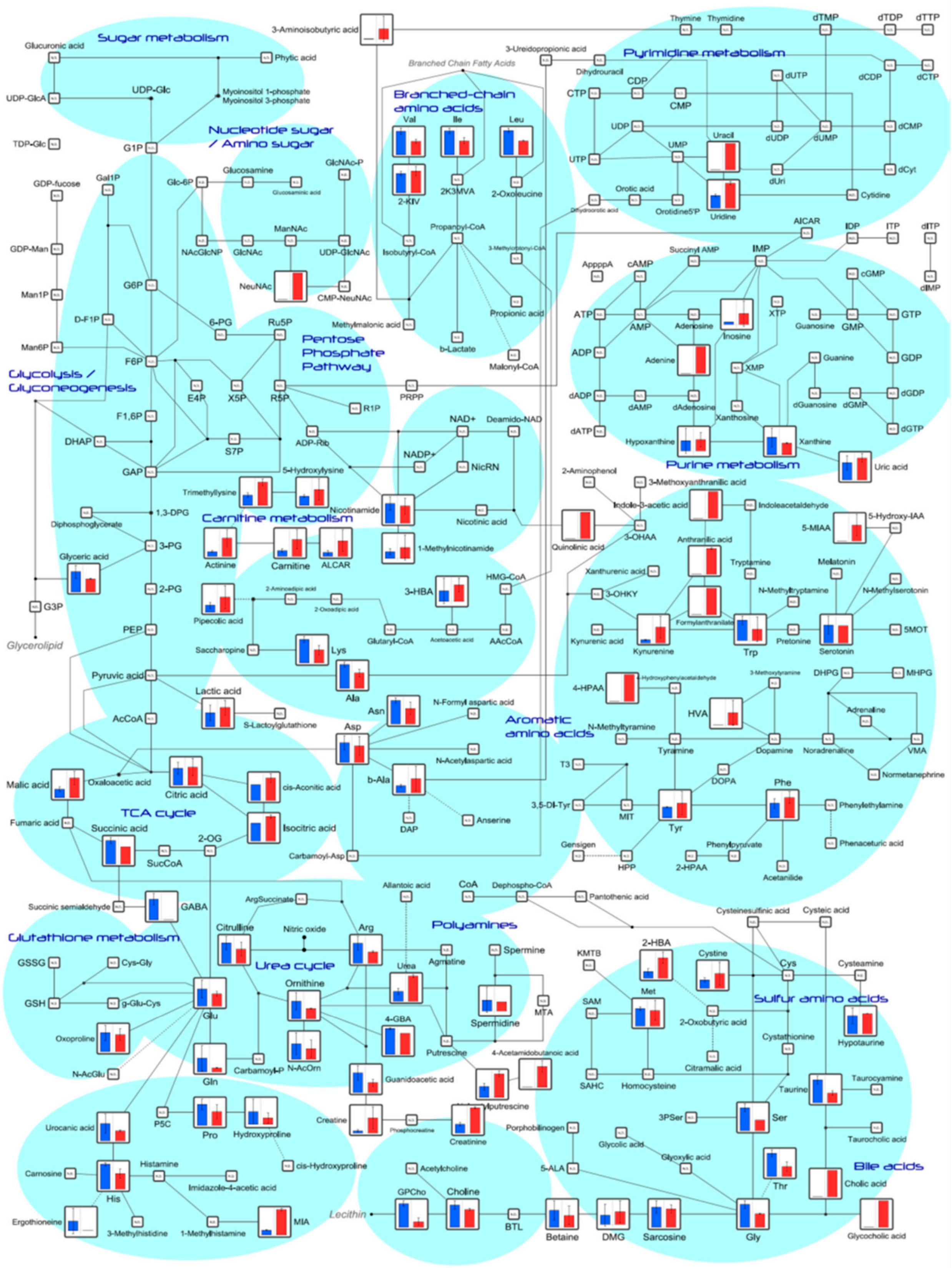

Figure S1. Total pathway map and kynurenine pathway map with the obtained metabolome data from exploratory metabolome profiling analysis (full version).

The metabolomics pathway map was created using the relative peak area of putative metabolites from seven serum samples, including from two subjects in the VAKI group (VT-1 and VT-3), two subjects in the infection subgroup (VN-1 and VN-2), and three HCs (H-1, H-2, and H-3) by the VANTED software. The blue and red boxes represent the mean relative peak area of the component in non-VAKI subjects and VAKI subjects, respectively.
